# Potential of Genome Editing to Capture Diversity From Australian Wild Rice Relatives

**DOI:** 10.3389/fgeed.2022.875243

**Published:** 2022-04-27

**Authors:** Muhammad Abdullah, Pauline Okemo, Agnelo Furtado, Robert Henry

**Affiliations:** ^1^ Queensland Alliance for Agriculture and Food Innovation, University of Queensland, Brisbane, QLD, Australia; ^2^ ARC Centre for Plant Success in Nature and Agriculture, University of Queensland, Brisbane, QLD, Australia

**Keywords:** gene editing, australian wild rice, CRISPR-Cas9, genetic diversity, novel alleles

## Abstract

Rice, a staple food worldwide and a model crop, could benefit from the introduction of novel genetics from wild relatives. Wild rice in the AA genome group closely related to domesticated rice is found across the tropical world. Due to their locality outside the range of domesticated rice, Australian wild rice populations are a potential source of unique traits for rice breeding. These rice species provide a diverse gene pool for improvement that could be utilized for desirable traits such as stress resistance, disease tolerance, and nutritional qualities. However, they remain poorly characterized. The CRISPR/Cas system has revolutionized gene editing and has improved our understanding of gene functions. Coupled with the increasing availability of genomic information on the species, genes in Australian wild rice could be modified through genome editing technologies to produce new domesticates. Alternatively, beneficial alleles from these rice species could be incorporated into cultivated rice to improve critical traits. Here, we summarize the beneficial traits in Australian wild rice, the available genomic information and the potential of gene editing to discover and understand the functions of novel alleles. Moreover, we discuss the potential domestication of these wild rice species for health and economic benefits to rice production globally.

## Introduction

The CRISPR-Cas system has quickly gained popularity as a strong and widely used tool for genome editing as compared to traditional inefficient and laborious random mutagenesis and screening methods (Ma X. et al., 2015; McCarty et al., 2020). The introduction of genome edits, like substitutions, insertions, and deletions, using the CRISPR-Cas 9 system, can speed up the breeding of plants including rice (Romero and Gatica-Arias, 2019). Australian wild rice represents an untapped source of important alleles that are missing from the rice gene pool (Henry et al., 2010). To ensure rice food security, it is necessary to increase productivity which relies on continuous genetic improvement (Brar and Khush, 2018; Henry, 2019). The wild rice species have higher drought, salinity, lodging, disease, and insect resistance than the most tolerant or resistant rice genotype. Additionally, they have unique traits such as acid soil tolerance, shade tolerance, high micronutrient content and are not only known to tolerate biotic and abiotic stress but also to exhibit extraordinary growth and development traits, such as profuse tillering and the existence of a salt gland that might be transferred to cultivated rice, increasing production and profitability (Henry, 2018; Moner and Henry, 2018).

The primary gene pool of rice comprises the *Oryza* A-genome species that are easily interfertile with rice (Wambugu et al., 2015). Previous research indicates two separate and unique perennial wild populations in tropical Australia (Brozynska et al., 2017; Moner et al., 2020), an *O. rufipogon*-like population, that has been referred to as Taxa-A, and *O. meridionalis*, including both perennial and annual forms and sometimes, referred to as Taxa-B. Genome analysis suggests that the *O. meridionalis* populations diverged from the lineage that became *O. sativa* approximately 3 Mya, while the Australian *O. rufipogon* like populations diverged approximately 1.6 Mya. The phylogenetic relationships between these species have been studied using both chloroplast and nuclear genome sequences (Wambugu et al., 2015; Brozynska et al., 2017). Taxa A (*O. rufipogon*-like taxa) has a chloroplast that is more similar to that of *O. meridionalis* and a nuclear genome that is more similar to that of *O. rufipogon* from Asia (Brozynska et al., 2017). A recent analysis of these taxa has confirmed that there is ongoing reticulate evolution, with rare hybrid plants being found in the wild (Hasan et al., 2022). *O. meridionalis* is the most distant species in the AA genome group that includes domesticated rice making it a significant resource for improving rice and studying rice evolution. In addition to being a source of slowly digestible starch and higher amylose content, its photosynthetic traits and abiotic stress tolerance make it an excellent candidate for use in the rice improvement (Scafaro et al., 2009; Tikapunya et al., 2017). Until recently, Australian wild rice was generally undisturbed by the impact of rice domestication, resulting in the persistence of wild Oryza in vast populations across a large area of northern Australia (Henry et al., 2010). These Australian Oryza may be critical in adapting rice to rapidly changing climate conditions and altering consumer preferences and needs. Moreover, recent data reveals that, even though the rice was first domesticated in Asia, Australian wild rice populations have provided genes to the domestication of rice (Huang et al., 2012; Fujino et al., 2019).

Seed shattering is a significant drawback affecting yield loss in both taxa of Australian wild rice. Gene editing using CRISPR-Cas to induce loss of function in shattering genes could allow rapid production of potentially new wild rice cultivars (Bohra et al., 2021). Advancement in genome and transcriptome sequencing has been a major contributor to improving gene target identification. The genomes of many wild rice species have been sequenced allowing the discovery of the genes responsible for desirable characteristics. The availability of these genetic resources is highly beneficial in supporting molecular breeding by horizontal transfer of key traits from wild species to cultivated rice.

**FIGURE 1:**
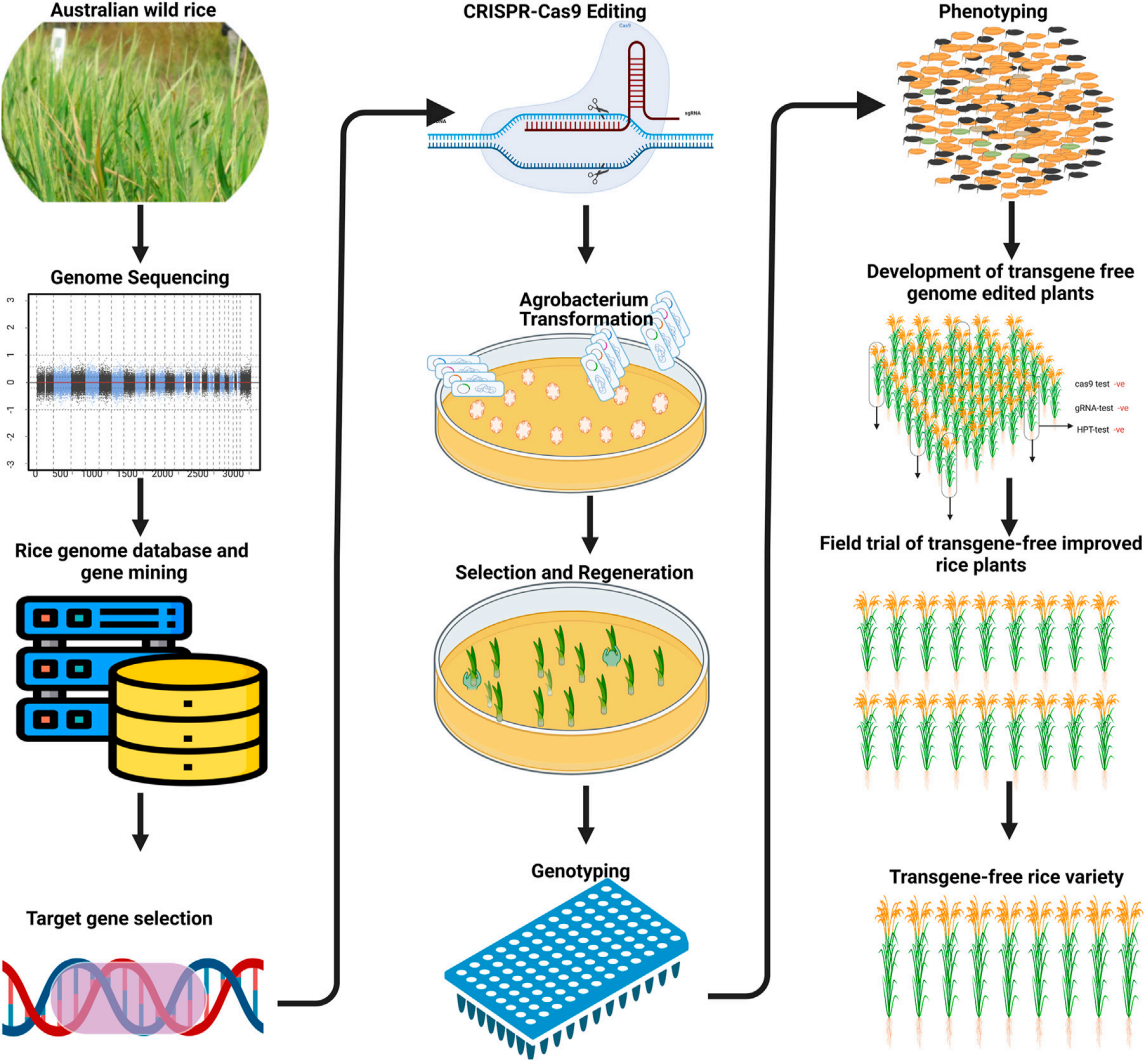
Schematic diagram shows the denovo domestication of Australian wild rice through genome editing.

In this review, we will discuss genome editing and how it has been used to capture diversity in rice (Figure 1). Furthermore, we will discuss how the function of novel alleles have been identified in domesticated rice using CRISPR/cas9 and how these studies can guide the identification of useful alleles in wild rice (especially in the Australian species) with the potential of being used in rice breeding.

## Gene Editing of Rice

Genome editing tools have broadened the range of options for rice research and improvement, giving scientists innovative ways to make new varieties that are more productive and better for the environment. The small size of the rice genome, high efficiency of transformation, abundance of genetic resources, and genomic synteny with other cereals provides an excellent model system for the study of functional genomics. In recent years, rice has been used to evaluate the efficacy of several genome editing methods, as well as to explore gene functions and their potential in the rice improvement (Li et al., 2012; Feng et al., 2013; Zafar et al., 2020) as briefly discussed below and highlighted in Table 1. CRISPR/Cas9-mediated editing of the *bsr-k1* gene produced higher-yielding rice plants resistant to leaf blast and bacterial leaf blight (Zhou et al., 2018). When *Bsr-d1*, *Pi21*, and *ERF922* were mutated using CRISPER/Cas-9 in all single and triple mutants of TGMS rice line (Indica thermosensitive genic male sterile) and longke638S (LK638S) were more resistant to rice blast than the wild type (Zhou et al., 2021). To find new sources of RTD (rice tungro disease) resistance, a CRISPR/Cas9 system was used to create mutations in the *eIF4G* gene in the RTSV-susceptible variety IR64, which is grown all over tropical Asia. *eIF4G* alleles with mutations in the SVLFPNLAGKS (mostly NL) close to the YVV residues were the only ones that were identified resistant (Macovei et al., 2018). Overexpression of *OsAAP3* in transgenic plants resulted in reduced bud outgrowth and rice tillering while *OsAAP3* RNAi slightly reduced the transport of amino acids, with lower concentrations of Arg, Lys, Asp, and Thr, but increased the number of bud outgrowth, tillers, grain production, and nitrogen usage efficiency (NUE). *OsAAP3* promoter sequences differed in Japonica and Indica rice, and expression was higher in Japonica, which had fewer tillers. CRISPR technology was used to create *OsAAP3* knockout lines in Japonica ZH11 and KY131 resulting in an increased grain yield (Lu et al., 2018).

**TABLE 1:** Summary of gene edited traits in rice.

Gene	Effect of Gene on plant	Genome-editing system	References
DST	Salinity tolerance, osmotic tolerance	CRISPR-Cas9	Santosh Kumar et al. (2020)
OsFWL4	Grain yield, plant architecture, number of tillers, flag leaf area, grain length	CRISPR-Cas9	Gao et al. (2020)
BADH2	Enhanced fragrance	CRISPR-Cas9	Ashokkumar et al. (2020)
OsSPL16/qGW8	Grain yield, grain weight, grain size	CRISPR-Cas9	Usman et al. (2020a)
Cytochrome P450, OsBADH2	Grain yield, grain size, aroma (2-acetyl-1-pyrroline (2AP) content)	CRISPR-Cas9	Usman et al. (2020b)
OsWaxy	Decrease in amylose content (glutinous rice)	CRISPR-Cas9	Huang et al. (2020)
OsMYB30	Cold tolerance	CRISPR-Cas9	Zeng et al. (2019)
OsALS	confers herbicide resistance	Base Editor and CRISPR-Cas9	Li et al. (2019a)
OsSPL14	gene for ideal plan architecture	Base Editor	Hua et al. (2019)
BBM1	enables embryo formation from a fertilized egg	CRISPR-Cas9	Khanday et al. (2019)
REC8, PAIR, OSD1, and MTL	for heterozygosity fixation and haploid induction	CRISPR-Cas9	Wang et al. (2019a)
SF3B1	confers resistance to splicing inhibitors	CRISPR-direct evolution	Butt et al. (2019)
SD1	Grain yield, plant architecture,semi-dwarf plants, resistance to lodging	CRISPR-Cas9	Hu et al. (2019)
Gn1a, GS3	Grain yield, panicle architecture, number of grains per panicle, grain size	CRISPR-Cas9	Shen et al. (2018)
eIF4G	Rice tungro spherical virus (RTSV)	CRISPR-Cas9	Macovei et al. (2018)
GS9, DEP1	Slender grain shape, less chalkiness	CRISPR-Cas9	Zhao et al. (2018)
OsPDS and OsSBEIIB	encode phytoene desaturase and starch branching enzyme	CRISPR-Cas12a	Li et al. (2018a)
OSCDC48	regulates senescence and cell death	Base Editor (C-to-T substitution)	Zong et al. (2018)
elF4G	candidate rice tungro disease resistance gene	CRISPR-Cas9	Macovei et al. (2018)
Gn1a, GS3	grain yield	CRISPR-Cas9	Shen et al. (2018)
Gn1a, DEP1	grain yield	CRISPR-Cas9	Huang et al. (2018)
PYL1, PYL4, PYL6	control plant growth and stress responses	CRISPR-Cas9	Miao et al. (2018)
OsFAD2-1	converts oleic acid into linoleic acid	CRISPR-Cas9	Abe et al. (2018)
OsGA20ox2	Grain yield, plant architecture, semi-dwarf plants, reduced, gibberellins and flag leaf length	CRISPR-Cas9	Shen et al. (2018)
OsAnn3	Response to cold tolerance	CRISPR-Cas9	Shen et al. (2017)
OsSAPK2	Reduced drought, salinity, and osmotic stress, tolerance; role of gene in ROS scavenging	CRISPR-Cas9	Lou et al. (2017)
SBE1, SBEIIB	control amylose contents	CRISPR-Cas9	Sun et al. (2017)
OsNramp5	metal transporter gene	CRISPR-Cas9	Tang et al. (2017)
SAPK2	functions in ABA-mediated seed dormancy	CRISPR-Cas9	Lou et al. (2017)
GW2, 5 and 6	Grain yield, grain weight	CRISPR-Cas9	Xu et al. (2016)
GW2/GW5/TGW6	Increased grain length and width	CRISPR-Cas9	Xu et al. (2016)
OsERF922	responsible for rice blast resistance	CRISPR-Cas9	Wang et al. (2016)
Badh2	control rice fragrance	CRISPR-Cas9	Shan et al. (2015)
LOXs	affect seed storability	TALEN-based genome editing	Ma et al. (2015a)
OsSWEET13	bacterial blight susceptibility genes	CRISPR-Cas9	Zhou et al. (2015)
ROC5, SPP, YSA	Disruption results in albino phenotype	CRISPR-Cas9	Feng et al. (2013)
OsSWEET14	bacterial blight susceptibility genes	CRISPR-Cas9	Jiang et al. (2013)

### Recent Advances in Editing Technology

The CRISPR-Cas9 system is mainly confined to genome editing at canonical NGG protospacer adjacent motif (PAM) sites. These sites are extremely important for nuclease identification, cleavage and efficient editing. Cas9 orthologs with changed PAM specificities have been discovered such as SaCas9 (*Staphylococcus aureus*) and Cas9-VQR (D1135V/R1335Q/T1337R) (Kleinstiver et al., 2015; Hu et al., 2016). Cas9-VQR has been designed to cleaves the sites containing a NGA PAM, however its editing efficiency was found to be insufficient in rice. To boost the VQR variant’s editing efficiency, the sgRNA structure was changed and significantly increased the editing efficiency (Hu et al., 2018). The CRISPR-SaCas9 toolkit was recently refined in rice by adding three important mutations (E782K/N968K/R105H) to improve the editing efficiency (Qin et al., 2019; Zafar et al., 2020). The editing efficiency of SaCas9 in the *PDS* and *DL* genes was determined via Agrobacterium-mediated transformation of Japonica rice. After mutagenesis, 34 out of 53 lines (64.2%) and 28 out of 36 (77.8%) lines had targeted mutations in the *PDS/T1* and *DL/T1* areas, respectively (Qin et al., 2019).

Cas9 with extended PAM SpCas9 (xCas9) and Cas9-NG (Cas9-NG) have also been tried in rice (Zhong et al., 2019) with xCas9 technology showing a better outcome in the rice genome editing (Wang J. et al., 2019; Endo et al., 2019). These enzymes can detect NG and GAA PAMs. The Cas9-NG also detects non-canonical PAM sites such as NCGAA and NG in addition to NCG (Ren et al., 2019; Zhong et al., 2019). These findings have broadened the breadth of rice genome editing.

Base editing is a novel approach to genome editing that enables irreversible base alterations at target loci without the use of double-stranded breaks or homology guided repair. (Hua et al., 2019). The combination of Cas9 nickase and cytidine deaminase enzymes allows for the creation of C to T or G to A substitutions anywhere in the genome (Komor et al., 2016; Mishra et al., 2018). For instance, the substitution of C-to-T in the *OsALS* gene resulted in an amino acid change at position 96 from alanine to valine conferred herbicide tolerance in *Oryza sativa* L (cv. Nipponbare) (Sun et al., 2016; Shimatani et al., 2017) (Table 1).

The tandem use of adenine and cytosine base editors in rice also shows their potential for use in the rice improvement (Hua et al., 2018). Human APOBEC3A and Cas9 nickase were used together to improve the efficiency of base editors (Zong et al., 2018). This fusion protein effectively converts cytidine to thymidine, allowing for larger editing frames, from 5 to 17 nucleotides in rice (Zong et al., 2018). Other recent examples of better base editing toolkits include (ABE)-nCas9 tool, SpCas9-NGv1, and ABE-P1S (Hao et al., 2019; Negishi et al., 2019; Hua et al., 2020). Although base editing is a highly effective method for inducing point mutation with high efficiency, base editors can’t generate exact indels, transversions, insertions, or avoid other mutations (Lin et al., 2020).

In contrast, prime editors have the ability to insert any of the 12 conceivable transition and transversion mutations as well as minor indels into the genome. Prime editing is a revolutionary method of genome editing (Anzalone et al., 2019). Instead of using a donor repair template, prime editing installs the desired modifications directly into the pegRNA sequence. Over the last few years, several attempts have been made to develop a reliable primary editing system in rice, with some success in creating herbicide-tolerant cultivars of rice (Li et al., 2020). Base and prime editing could contribute to domesticating Australian wild rice and significantly improving cultivated rice to overcome food security challenges.

### Applications of Gene Editing to Wild Rice Relatives

CRISPR-Cas technology allows for rapid *de novo* domestication of wild plant relatives. Traditional domestication requires considerable cross-breeding and selection of naturally occurring genetic alterations. Groundcherry (*Physalis pruinosa*) and wild tomato were recently *de novo* domesticated by utilizing genome editing (Li T. et al., 2018; Lemmon et al., 2018; Zhu and Zhu, 2021). Yu et al., 2021 outlined a *de novo* domestication strategy for *Oryza alta*, an allotetraploid rice with high biomass that is widely adapted to the environment (Yu et al., 2021). *Yu* et al., 2021 knocked out genes associated with seed shattering and awn length (*qSH1* and *An-1* orthologues), resulting in a considerably lower seed shattering rate and shorter awn length. To improve additional traits, they edited several orthologues of rice genes semi-dwarf stature (*SD1*), grain length and size (*GS3*), heading date (*Ghd7, DTH7*), and ideal plant architecture (*IPA1*) in *O. alta*. This remarkable study introduced a new era of rapid domestication of crops with desired traits by applying precise genome editing technologies. To domesticate a wild crop relative, it must have a well-sequenced genome and be amenable to tissue culture and transformation. The capacity to induce callus and regenerate plantlets is frequently a bottleneck to build a plant genetic transformation system. Only a few plant species, including a few *Oryza sativa* cultivars, have adequate and robust transformation procedures, several hurdles remain in applying genome editing to rice wild relatives.

## Australian Wild Rice

Henry et al., 2010 reported four Australian wild relatives *Oryza rufipogon* like population (Taxa-A), *Oryza meridionalis* like population (Taxa-B), *Oryza officinalis,* and *Oryza australiensis* (Henry et al., 2010; Brozynska et al., 2017). The characterization of unique wild rice species in Australia, via genetic and morphological investigation, has led to the discovery of novel Oryza gene pools (Waters et al., 2012; Sotowa et al., 2013; Brozynska et al., 2014). The AA genome species of most interest have been described above but the much more divergent *O. australiensis* is also of potential value in rice improvement. *Oryza australiensis,* the only known member of the E genome in the genus Oryza has unique characteristics such as an underground rhizome that a prospective source of novel genes for rice development because it allows plants to survive during the dry season (Henry et al., 2010). The relationships of *Oryza australiensis* with other species in the Oryza genus suggested that it may be useful in understanding the evolution of the Oryza genus. *Oryza australiensis* has a large and poorly characterised, with a high proportion of repeated sequences, making it challenging to study (Henry, 2018). In addition, the species shows outstanding grain properties, which suggests that it might potentially be used as a crop if domesticated (Tikapunya et al., 2016).

Genomic sequencing of these novel Australian wild rice species has been reported (Brozynska et al., 2017) but improved genome sequences are required to facilitate genome editing of rice to transfer their desirable traits.

### Potential Applications to Introgression of Genes From Australian Wild Rice

Biotechnological and genomic breakthroughs in rice genomic studies have created new prospects for improving rice germplasm with unique genetic features and better knowledge of rice gene activity. High-yielding improved rice varieties have been developed by applying traditional breeding procedures and manipulating the rice (*Oryza sativa*) gene pool resulting in better quality features. The cultivated rice gene pool has little genetic diversity hence interspecific hybridization could play a role in introducing economically important agronomic traits from wild to cultivated rice. However, due to incompatible obstacles, including pre-and postfertilization barriers, seed shattering, hybrid sterility, poor grain properties, and linkage drag, gene transfer from wild to domesticated species is challenging (Brar and Khush, 2018). Interspecific hybridization has enabled researchers to get and measure the genetic diversity of aliens from different Oryza genomes. Wild rice species have provided functional genes that make plants resistant to bacterial blight, tungro, brown planthoppers and acidic soils (Table 2).

**TABLE 2:** List of the key biotic stress resistance genes and QTLs identified within wild rice species.

Genes/QTLs	Marker	Inheritance	Wild species	References
Bacterial blight				
*xa45(t)*	LOC_Os08g42410 (STS)	Recessive	*O. glaberrima*	Neelam et al. (2020)
*xa32*	RM6293 and RM5926	Recessive	*O. australiensis*	Zheng et al. (2009)
*Xa27*	M964 and M1197	Dominant and cloned	*O. minuta*	Gu et al. (2004)
*Xa30*	RM1341, V88, C 189, 03STS	Dominant	*O. rufipogon*	Xuwei et al. (2007)
*qBBR5*	*RM7081–RM3616 5*		*O. meyeriana*	Chen et al. (2012)
Rice blast				
*Pi69(t)*	STS69-15-STS69-7 and RM20676	Dominant	*O. glaberrima*	Dong et al. (2020)
*qShB6*	RM3431		*O. nivara*	Eizenga et al. (2013)
*Pi57*	RM27892 and RM28093	Dominant	*O. longistaminata*	Xu et al. (2015)
*qBLAST8*	RM1148– RM210		*O. nivara*	Eizenga et al. (2013)
*Pi54rh*	*Pi54rh* Specific primer 625 bp	Dominant and cloned	*O. rhizomatis*	Das et al. (2012)
*Pi68*	SNP5 and RM14738	Dominant	*O. glumaepatula*	Devi et al. (2020)
Brown Planthopper (BPH)				
*Bph18*	BIM3-BN162	Dominant and cloned	*O. australiensis*	Ji et al. (2016)
*qBph4.2*	RM261-XC4–27		*O. australiensis*	Hu et al. (2015)
*Bph14*	SM1-G1318	Dominant and cloned	*O. officinalis*	Du et al. (2009)
*Wbph8*	R288-S11182	Dominant	*O. officinalis*	Tan et al. (2004)
*bph20(t)*	BYL7-BYL8	Recessive	*O. rufipogon*	Yang et al. (2011)
*Bph21(t)*	RM222-RM244	Dominant	*O. rufipogon*	Yang et al. (2011)
*bph22(t)*	RM8212-RM261	Recessive	*O. rufipogon*	Hou et al. (2011)
*bph23(t)*	RM2655-RM3572	Recessive	*O. rufipogon*	Hou et al. (2011)
*Bph27*	RM16846-RM16853	Dominant	*O. rufipogon*	Huang et al. (2013)
*Bph36*	RM16465-RM16502	Dominant	*O. rufipogon*	Li et al. (2019b)
*Bph38*	RM16563-RM16763	Dominant	*O. rufipogon*	Yang et al. (2020)

Italic value for scientific name and genes.

To capture useful genetic diversity, screening and phenotyping of many different accessions are very important. For example, only one *O. nivara* accession (IRGC101508) from India proved resistant to grassy stunt virus out of 6,000 cultivated and wild rice accessions examined.

### Potential Applications to the Domestication of Australian Wild Rice

Population growth and climate change threaten global agriculture productivity. To feed 10 billion people by 2050 is a massive challenge. To meet the world’s food needs and increase crop yields quickly, existing methods of domesticating crops are insufficient. Together with a deeper understanding of domestication’s genetic foundation, provided by pangenomes, recent advancements in gene editing technologies open the intriguing probability of developing novel crops by modifying few genes in wild species. Using a new platform for domestication, it may be possible to convert crop wild relatives quickly and precisely into economically desirable crops while keeping some of the beneficial resilience and nutritional properties that have been lost during domestication and breeding.

Australian wild rice has many unique and novel traits that can feed the future population. Australian wild rice domestication can potentially be achieved by following and optimizing the *de novo* route highlighted by Li’s group; the development of a high-performance transformation system, putting together and annotating a high-quality reference genome, and editing several genes that are important for domestication, e.g., shattering, awn length, panicle architecture and nutritional benefits to improve a variety of features. In this way, genome engineering might be used to generate nutritionally and climate-smart crops from the start in a wide range of crops currently used for human consumption, food production, animal feed, or biofuel.

## Future Prospects

Traditionally, domestication of wild plants into commercial crops took hundreds or even thousands of years, but newly emerging genome editing technologies enable this to be accomplished in a few generations (Van Tassel et al., 2020). As a result, effective genome editing techniques are critical for accelerating the speed of domestication. Only the *O. sativa* subspecies japonica and indica have been successfully transformed using Agrobacterium-mediated transformation systems (Hiei et al., 1994). To determine the most promising starting material, priority must be given to callus induction and regeneration capacities with suitable biomass traits and stress tolerance etc. During the domestication, traits that were good for farming instead of natural growth were chosen and improved, such as grain size, hull colour, erect growth, shattering, pericarp colour and awn etc (Chen et al., 2019). Many traits of Australian wild rice species are similar to those of the wild ancestors of the present cultivars because they are closely related. Identifying the wild rice homologs of the domestication-related genes from domesticated rice is the first step, for example *qSH1*gene homolog for seed shattering, *Bh4* homolog gene for hull colour, *An-1* and *An-2* for awn length, *Rc* for pericarp colour, *OsLG1* for panicle shape, and *GW5* for grain width. Editing these homologs genes by utilising a CRISPR/Cas9-mutagenesis technique may genuinely achieve quick domestication of Australian wild rice.

Most crop improvements have involved targeted editing and transformation, which require the efficient transformation and precise large-scale genome editing system. For example, RNA viral vectors, may infect plants and deliver gene-editing reagents to the germline, inducing hundreds to thousands of different mutations. Using developmental regulators, altered somatic cells can generate meristems that produce seed-bearing branches, boosting productivity and minimizing timeframes (Nasti and Voytas, 2021). These and other techniques will allow faster breeding, domestication of Australian wild rice, and metabolic reengineering than previously conceivable. So, developing an efficient transformation and genome editing system for Australian wild rice is very important.

Furthermore, Australian wild rices have beneficial traits including biotic and abiotic stress tolerance that can be used in breeding programs for improved yield. Studies on the loss or gain of function of the genes associated with these traits need to be conducted to definitively understand their mechanisms and potentially edit them into cultivated rice varieties.
